# The cytotoxic and stress responses of human trabecular meshwork cells treated with triamcinolone acetonide

**Published:** 2008-01-22

**Authors:** Dan Yi Wang, Bao Jian Fan, Gary Y. H. Yam, Dennis S. C. Lam, Chi Pui Pang

**Affiliations:** Department of Ophthalmology & Visual Sciences, the Chinese University of Hong Kong, Hong Kong, China

## Abstract

**Purpose:**

To evaluate the cytotoxic effect of triamcinolone acetonide (TA) on cultured human trabecular meshwork (TM) cells.

**Methods:**

TA (0.1 mg/ml, 1 mg/ml) or the vehicle (benzyl alcohol, 0.0025%, 0.025%) was added to human TM cell cultures on day 0 and collected subsequently on day 1, 3, or 5. The amount of cell proliferations with or without TA treatment was measured using the 3-(4,5-dimethylthiazol-2-yl)-2,5-diphenylterazolium bromide (MTT) assay. All samples were read in triplicate (n=4 in all cases). By using real-time quantitative polymerase chain reaction (PCR), gene expression levels of *c-fos*, *c-jun*, *caspase-3*, *c-myc*, and *p53* were determined after TA treatments at 0 min, 10 min, 20 min, 30 min, 50 min, 80 min, 2 h, 12 h, 24 h, and 48 h. Unpaired *t*-test was used to test the drug and concentration effects of TA, ANOVA was used to test the time effects of TA, and the Bonferroni test was used to correct multiple comparisons. Apoptosis of TM cells as a result of TA treatment were assessed by the terminal uridyl nick end labeling (TUNEL) assay.

**Results:**

Both concentrations of TA caused a significant reduction in the number of human TM cells as early as day 1 and across five days of the treatment period. Significantly increased expressions of *c-jun*, *c-fos*, *c-myc*, *p53*, and *caspase 3* were observed at different time points after both 0.1 mg/ml and 1 mg/ml TA treatment. Significantly increased apoptotic cells were observed after TA treatment for three days.

**Conclusions:**

Our results showed that TA was cytotoxic to human TM cells in culture and the presence of TA caused apoptotic cell death. It gave evidence that the underlying mechanism of TA caused ocular hypertension and may be associated with necrosis and apoptosis of the TM cells.

## Introduction

Triamcinolone acetonide (9α-fluoro-16α-hydroxyprednisolone, TA) is commonly used through intravitreal injections (IVTA) for macular diseases such as uveitis [1,2], macular edema secondary to retinal vascular disease [3,4], intraocular proliferations such as proliferative vitreoretinopathy [5], and choroidal neovascularization from age-related macular degeneration [6]. IVTA provides a direct route to delivering the drug to the tissue cells of the posterior segment. However, adverse events associated with the use of IVTA have also been reported including intraocular pressure (IOP) elevation, pseudoendophthalmitis, endophthalmitis, and cataractogenesis [7–9]. However, in rabbits, IVTA caused relatively little or no retinal toxicity after seven days, according to electroretinography [10]. Two recent reports also revealed no electroretinographic or histological disruptions to the rabbit retina after 28 days and 12 weeks [11,12].

One major side effect of IVTA is the induced elevation of IOP. A dosage of 25 mg led to a secondary ocular hypertension in about 50% of the treated eyes, and the rise of IOP was reversible around six months after the injection [3]. Meta-analysis of a series of 272 patients showed that 20 mg IVTA caused IOP elevation after one week, before subsiding to the baseline level after eight to nine months [13]. Another case series of 43 patients received a lower dose of 4 mg IVTA and there was no effect on IOP within seven days [14]. Although the intraocular concentration of TA may fall below the therapeutic range before 90 days, persistence of even a trace amount may lead to prolonged ocular hypertension occasionally seen in some patients [15]. The human trabecular meshwork (TM) in the chamber angle accounts for most aqueous outflow resistance in the anterior chamber of the human eye and participates in the regulation of intraocular pressure. It is believed that the interplay between the TM cells and the surrounding extracellular matrix is responsible for maintaining the resistance necessary for the preservation of the aqueous outflow pathway [16]. Like many other corticosteroids, TA might affect the TM structural framework, inhibit protease activities, or increase protein expression that causes disruption of the aqueous outflow [17]. Since TM cells are in constant contact with the aqueous humor, TA in the aqueous humor may have direct biologic effects on the TM tissue cells. We have found cytotoxicity caused by TA on photoreceptors and retinal pigment epithelial (RPE) cells in culture [18]. TA also decreased the expression of vascular endothelial growth factor, an angiogenic agent, but increased expression of the angiogenic inhibitor, pigment epithelial derived factor, in both ARPE19 and human umbilical vein endothelial cells [19]. Recently, we found the presence of TA in the aqueous humor of rabbits given 12 mg IVTA [20]. The TA presence in the aqueous humor of patients given IVTA was inconsistent [21]. Deposition of the extracellular matrix was likely to occur in two patients given 4 mg IVTA [22]. In this study, we examined whether TA causes toxic and stress responses of cultured human TM cells.

## Methods

### Trabecular meshwork cell culture and triamcinolone acetonide treatment

A human trabecular meshwork cell line, established from trabecular specimens obtained postmortem from a patient with no personal or family history of glaucoma [23], was courteously provided by Dr. Thai Nguyen, University of California San Francisco (San Francisco, CA). Cell culture reagents, fetal bovine serum (FBS), dimethylsulfoxide (DMSO), penicillin G, streptomycin, phosphate-buffered saline (PBS), Dulbecco’s Modified Eagle Media (DMEM), and trypsin were purchased from Invitrogen Co. (Carlsbad, CA). Containers were from Corning Glass (Acton, MA). TM cells were propagated in DMEM containing 10% FBS, 100 U/ml penicillin G, and 100 μg/ml streptomycin sulfate. TM cell suspensions with cell volume of 1×10^4^ were seeded onto 100×20 mm tissue culture plates. The culture was maintained in a humidified 5% CO_2_ environment at 37 °C. All cells within the same 23^rd^ passage were grown to 80% confluence for TA treatment. The cultures of TM cells with 80% confluence were adapted into fresh culture medium 12 h before the addition of drugs. TA (Kenacort-A, Bristol-Myers-Squibb, New York, NY) was serially diluted in a culture medium to appropriate concentrations. The concentrations used were derived from the known concentrations used in experimental and clinical settings [4,18]. 0.1 mg/ml TA contained 0.0025% benzyl alcohol (Sigma-Aldrich, Munich, Germany) and 1 mg/ml TA contained 0.025% benzyl alcohol. TA (0.1 mg/ml, 1 mg/ml) or the vehicle benzyl alcohol (0.0025%, 0.025%) were well mixed and then added to the TM cells.

### MTT-cell proliferation assay

The amount of cell proliferations was determined using the 1-(4,5-dimethylthiazol-2-yl)-2,5-diphenyltetrazolium bromide (MTT) assay. Cells were washed with PBS. MTT at 0.5 mg/ml in a serum-free medium was then added to the cultures and incubated for 3 h at 37 °C in a 5% CO_2_ environment. Formazan extraction was performed with isopropanol, and the quantity was determined colorimetrically by using a spectrophotometer Powerwave XS at λ=570 nm with the correction of interference at 690 nm. All four replicated samples per group were read in triplicate. The MTT assay was performed one, three, and five days after the initial TA exposure. The results were expressed as units of absorbance of MTT at 570 nm ±SD.

**Figure 1:**
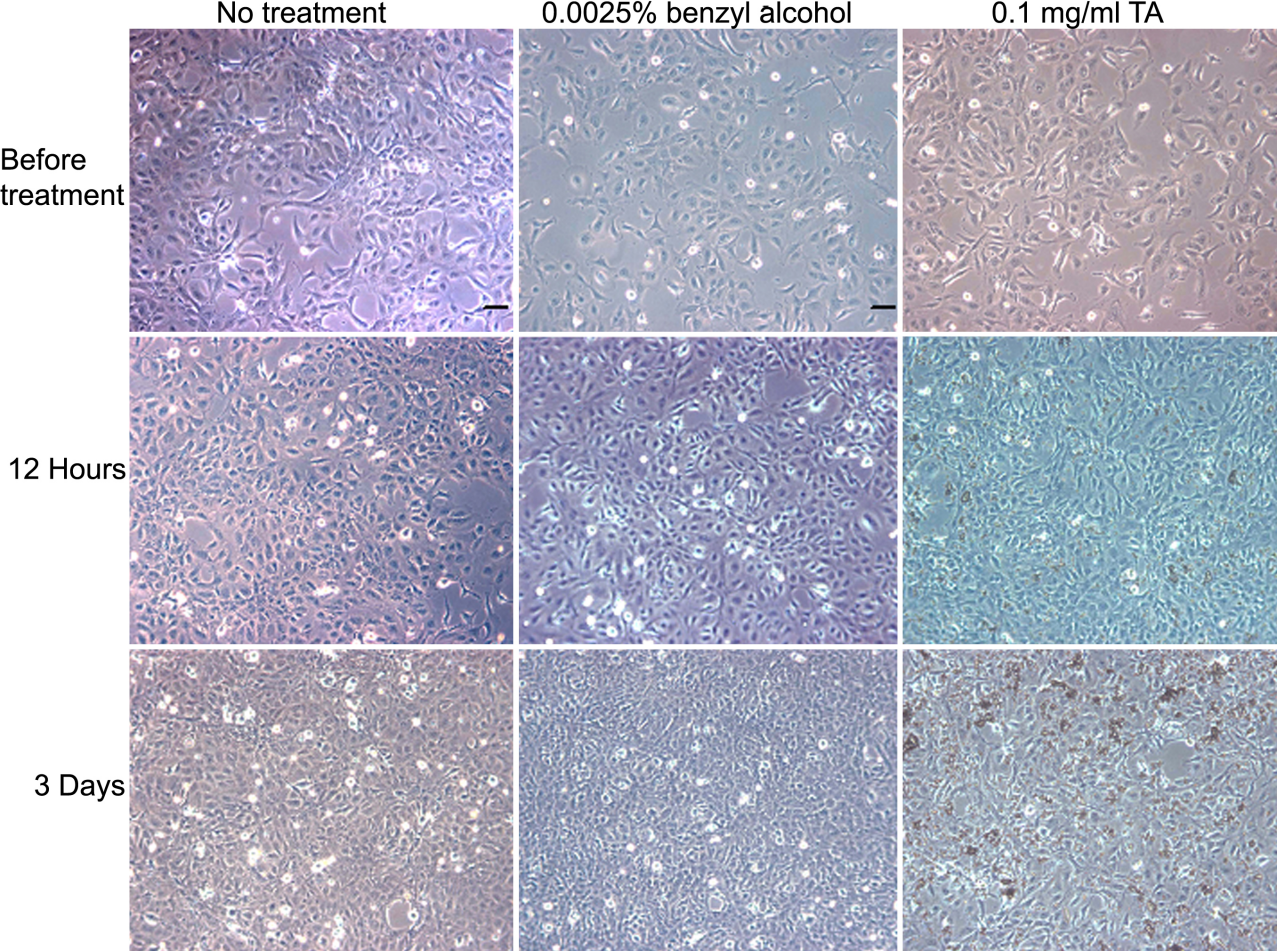
Phase-contrast micrographs showing human trabecular meshwork cells in 0.1 mg/ml TA (triamcinolone acetonide). In the presence of 0.1 mg/ml TA, a large number of randomly distributed TA particles could be seen on the top of the TM cells. Compared to no treatment and 0.0025% benzyl alcohol treatment, there was progressive reduction of cell numbers, and the TM cells became more oval and shrank after 0.1 mg/ml TA treatment. Scale bar: 50 μm.

### Gene expression study

The TM cells after exposure to TA or the vehicle benzyl alcohol were collected at 0 min, 10 min, 20 min, 30 min, 50 min, 80 min, 2 h, 12 h, 24 h, and 48 h for RNA extraction and real-time quantitative PCR (RT-qPCR). Every time point was measured in triplicate. Total RNA was extracted with RNeasy Mini Kit (Qiagen, Hilden, Germany) according to the manufacturer’s protocol. Briefly, cells were lysed in a lysis buffer containing 1% β-mercaptoethanol (Sigma, St. Louis, MO) and then passed through a separation column (QIAShredder; Qiagen). RNA samples were quantified with the NanoDrop® ND-1000 (NanoDrop Technologies, Wilmington, DE), and 500 ng of total RNA was used for reverse-transcription with 3 μg/μl random primer p[dN]6 (Roche Diagnostics, Mannherim, Germany) and a reverse transcriptase kit with a RNase inhibitor (Superscript III Reverse Transcriptase Kit and RNase OUT inhibitor; Invitrogen).

The amount of cDNA corresponding to 25 ng of RNA was selected and amplified with the primer pairs as previously reported [9]. The sequences of intron-spanning primers were as follows: *GAPDH* forward, 5′-GAA GGT GAA GGT CGG AGT-3′ and reverse, 5′-GAA GAT GGT GAT GGG ATT TC-3′; *c-jun* forward, 5′-GTG ACG GAC TGT TCT ATG ACT G-3′ and reverse, 5′-GGG GGT CGG CGT GGT GGT GAT G-3′; *c-fos* forward, 5′-AGA CAG ACC AAC TAG AAG ATG A-3′ and reverse, 5′-AGC TCT GTG GCC ATG GGC CCC-3′; *caspase 3* forward, 5′-TAT TCT TGG GGA AAT TCA AAG GAT-3′ and reverse, 5′-AAA GTA GCG TCA AAG GAA AAG GAC-3′; *p53* forward, 5′-TTG CCG TCC CAA GCA ATG GAT GA-3′ and reverse, 5′ΤCT GGG AAG GGA CAG AAG ATG AC-3′. RT-qPCR was performed using SYBR Green PCR master mix (BioRad Laboratories, Heracles, CA). ABI PRISM 7000® Sequence Detection System was used for real-time PCR (Applied Biosystems, Foster City, CA). The thermocycler parameters were 95 °C for 2 min followed by 40 cycles of 95 °C for 15 s and 60 °C for 1 min. The relative quantification was normalized to the *GAPDH* expression level. The mean Ct value (threshold cycle, cycle at which the increase in signal associated with exponential growth of PCR product was first detected) of the TA-treated sample was compared to that of the untreated control sample using the Ct value of *GAPDH* as an internal control. ΔCt was the difference in Ct values derived from the target gene (in each sample assayed) and *GAPDH* while ΔΔCt represented the difference between the paired samples. The n-fold differential ratio was expressed as 2^-ΔΔCt^. All data were expressed as mean±SD.

**Figure 2:**
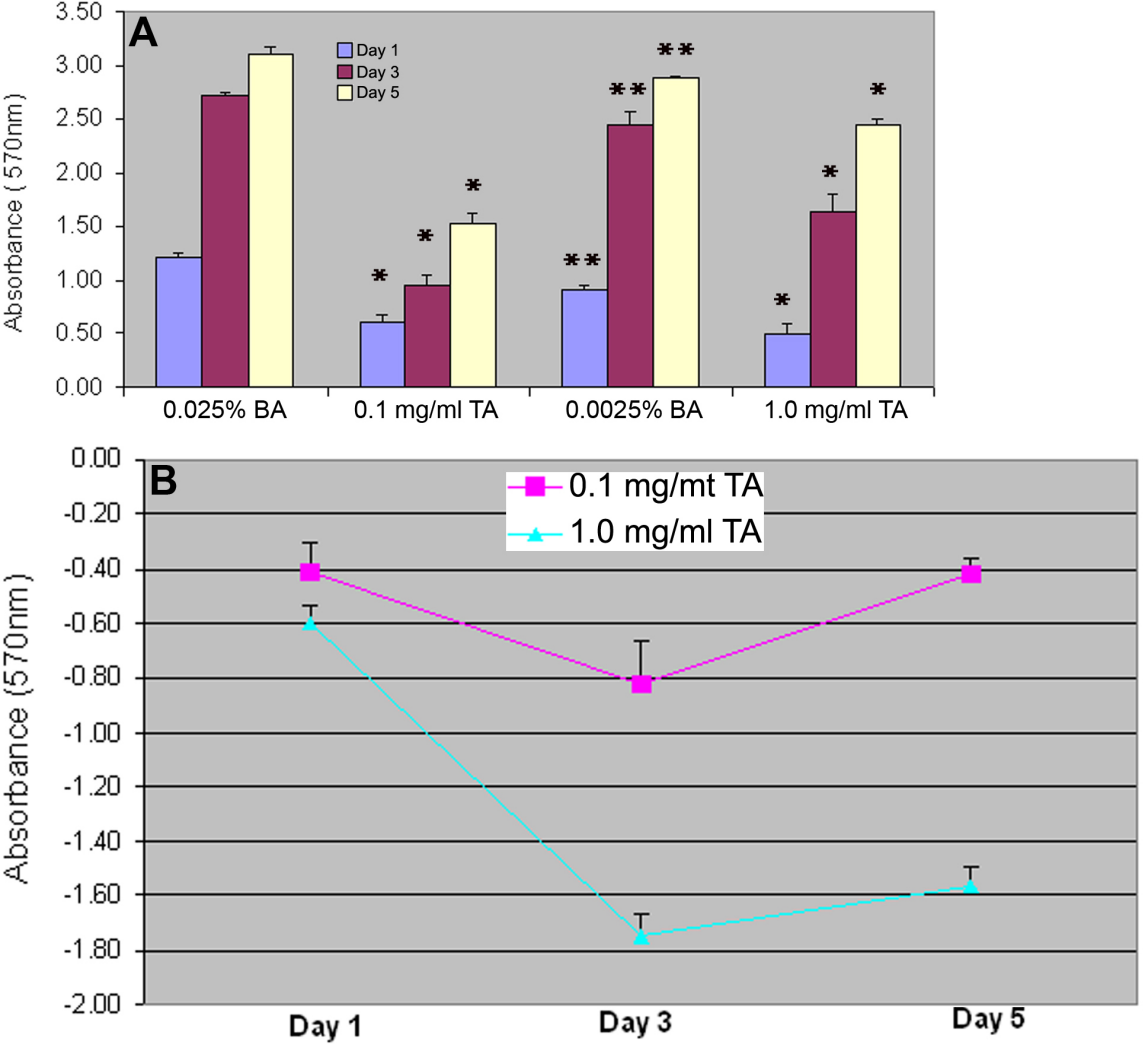
Proliferation of human trabecular meshwork cells in the presence of various concentrations of triamcinolone acetonide and over a five-day treatment period. **A: **The asterisk indicates that both concentrations of TA caused significant reduction in the number of TM cells as early as day 1 and across 5 days of the treatment period (p<0.01). The double asterisk means that there was a significant difference in the number of TM cells between 0.025% and 0.0025% benzyl alcohol in the culture (p<0.005). **B:** After eliminating the benzyl alcohol effect, there was no significant difference in cell proliferation between 0.1 mg/ml TA and 1 mg/ml TA one day after treatment (p=0.058) while a significant difference in cell proliferation was observed between the two concentrations of TA treatment on day 3 (p=0.003) and on day 5 (p<0.0005).

### Detection for apoptosis

The TM cells were examined for apoptosis 12 h as well as one, three, and five days after TA (0.1 and 1 mg/ml) and vehicle treatment (benzyl alcohol, 0.0025%, 0.025%). The cells were fixed and permeabilized. Apoptosis was determined using the terminal uridyl nick end labeling (TUNEL) in situ cell death detection TMR kit using terminal uridyl nick end labeling (Roche Diagnostics, Mannherim, Germany) according to the supplier's protocol. After nuclear staining by DAPI, the samples were examined by fluorescence microscopy (DMRB; Leica, Wetzlar, Germany) equipped with the Spot RT color system (Diagnostic Instruments, Sterling Heights, MI). The apoptosis percentage due to TA was the apoptotic TM cell number divided by the total TM cell number after adjustment of the vehicle (benzyl alcohol) effect.

### Statistical analysis

Unpaired *t*-test was used to test the drug and concentration effects of TA whereas ANOVA was used to test the time effect of TA. The Bonferroni test was applied to correct multiple comparisons. p<0.05 was considered to be statistically significant.

## Results

### Cytotoxicity of triamcinolone acetonide in trabecular meshwork cells

In the presence of 0.1 mg/ml TA, a large number of randomly distributed TA particles could be seen on top of the TM cells (Figure 1), which did not show morphological changes under light microscopy. When the concentration was increased to 1 mg/ml, no usable images could be obtained because the entire field of view was virtually covered by TA particles. The MTT experiments showed that both concentrations of TA caused a significant reduction in the number of TM cells as early as day 1 and throughout the five-day study period (Figure 2A). After eliminating the benzyl alcohol effect, there was no significant difference in cell proliferation between the two concentrations of TA after three days (p=0.003) and five days (p<0.0005; Figure 2B). At 1 mg/ml, TA caused a significant reduction in the number of TM cells on day 3 (p<0.0005) and on day 5 (p<0.0005) (Figure 2B). At 0.1 mg/ml, TA caused a significant reduction in the number of TM cells between day 1 and day 3, and there was a significant increase between day 3 and day 5 (p=0.018; Figure 2B). There was significant interaction between the concentration and time effects (p<0.0005).

### Gene expression study

**Figure 3:**
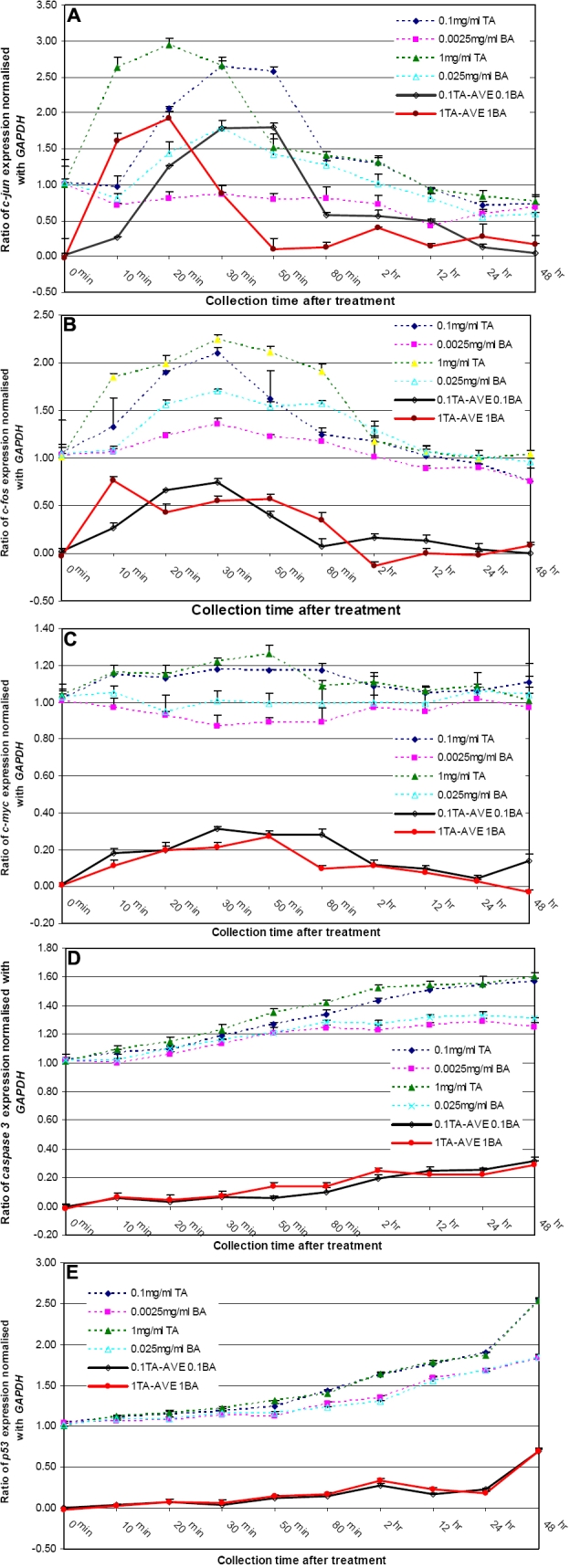
Changes in gene expression of *c-jun*, *c-fos*, *c-myc*, *caspase 3*, and *p53* after triamcinolone acetonide treatment. **A**: For 1 mg/ml TA, the change of c-jun expression peaked at 20 min and the change kept a significant increase between 10 min and 30 min. For 0.1 mg/ml TA, c-jun expression peaked at 50 min and kept significantly increasing between 10 min and 12 h. The changes in gene expression were concentration dependent between the 10 min-12 h interval. Between 10 and 20 min, the c-jun expression change was higher for 1 mg/ml TA treatment. However, the c-jun expression change was higher for 0.1 mg/ml TA treatment between 30 min and 12 h. **B**: For 1 mg/ml TA, the change of c-fos expression peaked at 10 min and the change kept significant elevation between 10 min and 80 min. For 0.1 mg/ml TA, there was no significant change in gene expression across various time points (p>0.05). Compared to 1 mg/ml TA, the changes in gene expression were higher for 0.1 mg/ml TA treatment between 20 min to 30 min. **C**: The change of c-myc expression peaked at 30 min and 50 min for 0.1 mg/ml TA and 1 mg/ml TA treatment, respectively. The gene expression change kept a significant increase between 10 min and 2 h for both TA concentrations. Compared to 1 mg/ml TA, the change in gene expression was higher for 0.1 mg/ml TA treatment at 10 min, 30 min, 80 min and 48 h. **D**: The change of caspase 3 expression for both concentrations of TA maintained a significant increase between 50 min and 48 h. Compared to 0.1 mg/ml TA, the change was higher for 1 mg/ml TA treatment only at 50 min. **E**: The change of p53 expression for both concentrations of TA increased significantly from 50 min to 48 h. The change in gene expression was concentration dependent between 2 h and 48 h. The expression change was greater for 1 mg/ml TA between 2 h and 12 h while the change was greater for 0.1 mg/ml between 24 h and 48 h. Dotted lines represent initial gene expression levels before eliminating benzyle alcohol (BA) effect from TA. Solid lines represent subtracted gene expression levels after eliminating BA effect from TA.

For all five genes investigated in the present study including *c-jun*, *c-fos*, *c-myc*, *p53*, and *caspase 3*, the mRNA expression levels were significantly elevated in the TA-treated TM cells compared to corresponding benzyl alcohol-treated TM cells. However, except for *p53*, there were significantly different gene expressions of *c-jun*, *c-fos*, *c-myc,* and *caspase 3* between 0.025% and 0.0025% benzyl alcohol treatment (Table 1, Figure 3). Therefore, we eliminated the benzyl alcohol effect by subtracting the gene expression value of each gene induced by benzyl alcohol from that induced by TA when we analyzed the concentration and time effects of TA treatment (Figure 3).

**Table 1:** Drug effects of gene expression of c-jun, c-fos, c-myc, caspase 3 and p53

Gene	Treatment	0 min	10 min	20 min	30 min	50 min	80 min	2 h	12 h	24 h	48 h
*c-jun*	0.025% BA versus 0.0025% BA	0.88	0.051	0.003	<0.0005	0.003	0.009	0.105	0.002	0.602	0.059
*c-jun*	0.1 mg/ml TA versus 0.0025% BA	0.886	<0.0005	<0.0005	<0.0005	<0.0005	0.004	0.003	0.001	0.073	0.571
*c-jun*	1 mg/ml TA versus 0.025% BA	0.891	<0.0005	<0.0005	0.001	0.452	0.051	<0.0005	0.361	0.075	0.080
*c-fos*	0.025% BA versus 0.0025% BA	0.883	<0.0005	<0.0005	<0.0005	<0.0005	0.001	0.274	0.011	0.044	0.029
*c-fos*	0.1 mg/ml TA versus 0.0025% BA	0.960	<0.0005	<0.0005	<0.0005	0.149	0.423	0.368	0.114	0.358	0.969
*c-fos*	1 mg/ml TA versus 0.025% BA	0.660	<0.0005	0.002	<0.0005	<0.0005	0.002	0.245	0.933	0.607	0.072
*c-myc*	0.025% BA versus 0.0025% BA	0.715	0.090	0.490	0.005	<0.0005	0.461	0.152	0.060	0.070	0.006
*c-myc*	0.1 mg/ml TA versus 0.0025% BA	0.596	0.005	0.004	<0.0005	<0.0005	0.001	0.005	0.005	0.070	0.003
*c-myc*	1 mg/ml TA versus 0.025% BA	0.814	0.804	0.005	0.001	<0.0005	0.442	0.008	0.007	0.065	0.081
*caspase 3*	0.025% BA versus 0.0025% BA	0.902	0.683	0.089	0.070	1.000	0.041	0.154	0.024	0.099	0.033
*caspase 3*	0.1 mg/ml TA versus 0.0025% BA	1.000	0.054	0.114	0.024	0.04	0.01	0.001	<0.0005	0.002	<0.0005
*caspase 3*	1 mg/ml TA versus 0.025% BA	0.692	0.675	0.087	0.027	0.006	0.001	<0.0005	<0.0005	0.002	<0.0005
*p53*	0.025% BA versus 0.0025% BA	0.305	0.21	0.709	0.516	0.152	0.057	0.057	0.51	0.189	0.718
*p53*	0.1 mg/ml TA versus 0.0025% BA	0.678	0.067	0.059	0.25	0.003	0.001	<0.0005	<0.0005	<0.0005	<0.0005
*p53*	1 mg/ml TA versus 0.025% BA	0.219	0.065	0.026	0.1	0.001	0.001	<0.0005	0.007	<0.0005	<0.0005

The gene expressions of all five genes were significantly elevated in the TA-treated TM cells compared to corresponding benzyl alcohol-treated TM cells. However, except for p53, there were significantly different gene expressions of c-jun, c-fos, c-myc and caspase 3 between 0.025% and 0.0025% benzyl alcohol treatment. The numbers are the p values for the drug effects of gene expressions. BA indicates benzyl alcohol.

For *c-jun*, the change in gene expression peaked at 20 min when TM cells were treated with 1 mg/ml TA and the change kept a significant increase between 10 min and 30 min. When TM cells were treated with 0.1 mg/ml TA, *c-jun* expression peaked at 50 min and kept significantly increasing between 10 min and 12 h. The changes in gene expression were concentration dependent between the 10 min-12 h interval. Between 10 and 20 min, the *c-jun* expression change was higher for 1 mg/ml TA treatment. However, the *c-jun* expression change was higher for 0.1 mg/ml TA treatment between 30 min and 12 h (Figure 3A).

For *c-fos*, the change in expression peaked at 10 min when TM cells were treated with 1 mg/ml TA, and the change kept significant elevation between 10 min and 80 min. When TM cells were treated with 0.1 mg/ml TA, there was no significant change in gene expression across various time points (p>0.05). When compared to 1 mg/ml TA treatment, the changes in gene expression were higher for 0.1 mg/ml TA treatment between 20 min and 30 min. (Figure 3B).

For *c-myc*, the change in expression peaked at 30 min and 50 min for 0.1 mg/ml TA and 1 mg/ml TA treatment, respectively. The gene expression change kept a significant increase between 10 min and 2 h for both TA concentrations. When compared to 1 mg/ml TA treatment, the change in gene expression was higher for 0.1 mg/ml TA treatment at 10 min, 30 min, 80 min, and 48 h (Figure 3C).

For *caspase 3*, the change in gene expression for both concentrations of TA maintained a significant increase between 50 min and 48 h. When compared to 0.1 mg/ml TA, the change in gene expression was higher for 1 mg/ml TA treatment only at 50 min (Figure 3D).

For *p53*, the change in expression for both concentrations of TA increased significantly from 50 min to 48 h. The change in gene expression was concentration dependent between 2 h and 48 h. The expression change was greater for 1 mg/ml TA between 2 h and 12 h while the expression change was greater for 0.1 mg/ml TA between 24 h and 48 h (Figure 3E).

### Apoptosis of trabecular meshwork cells after triamcinolone acetonide treatment

**Figure 4:**
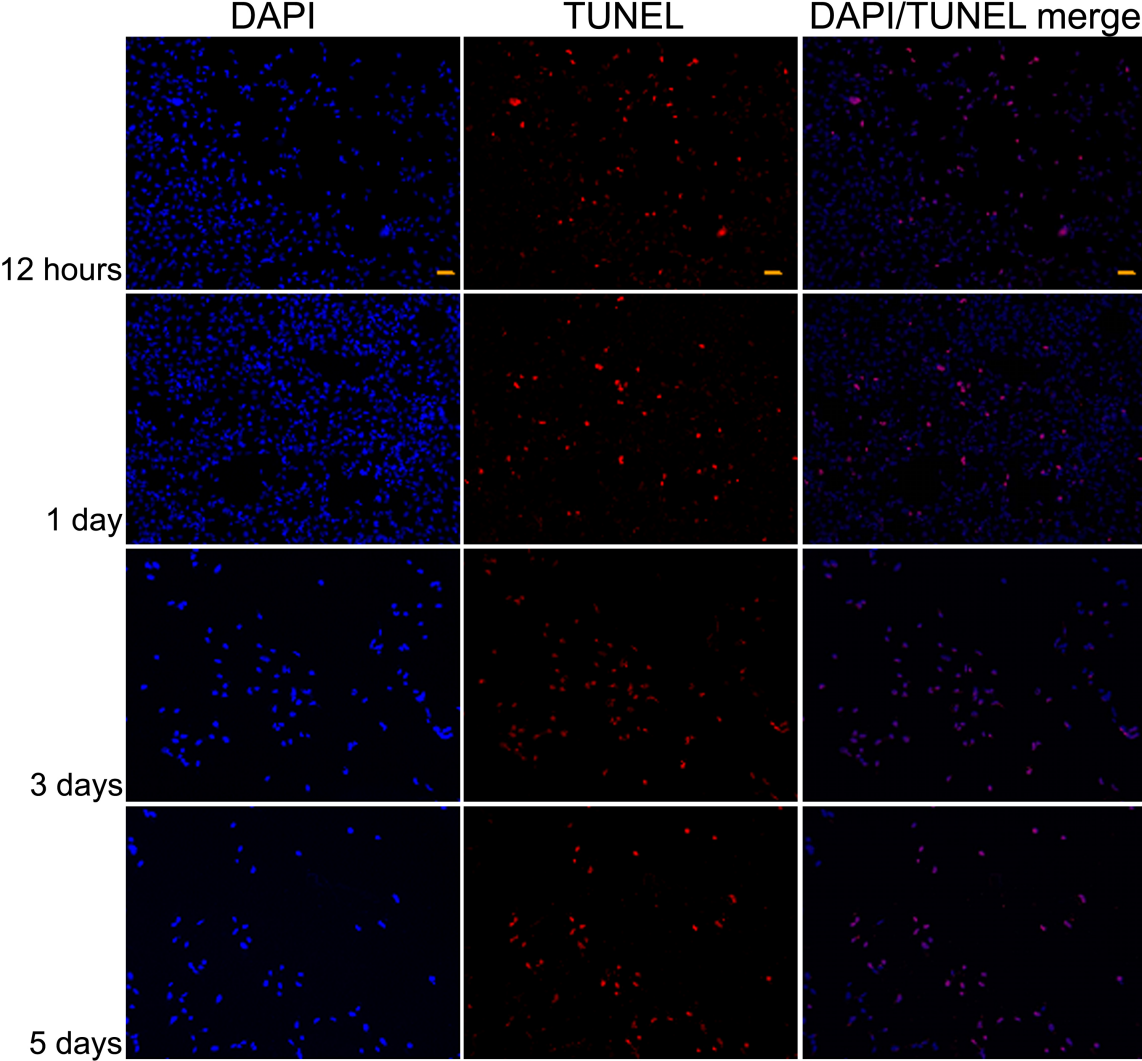
Apoptotic trabecular meshwork cells after 0.1 mg/ml triamcinolone acetonide treatment. Apoptotic nuclei of TM cells were detected 12 h as well as 1, 3, and 5 days after 0.1 mg/ml TA treatment. The slide was viewed with two filters. Stain red was viewed using a fluorescein filter to visualize end-labeled cells. Stain blue was viewed with a DAPI filter to visualize the entire cell population. Scale bar: 50 μm.

**Figure 5:**
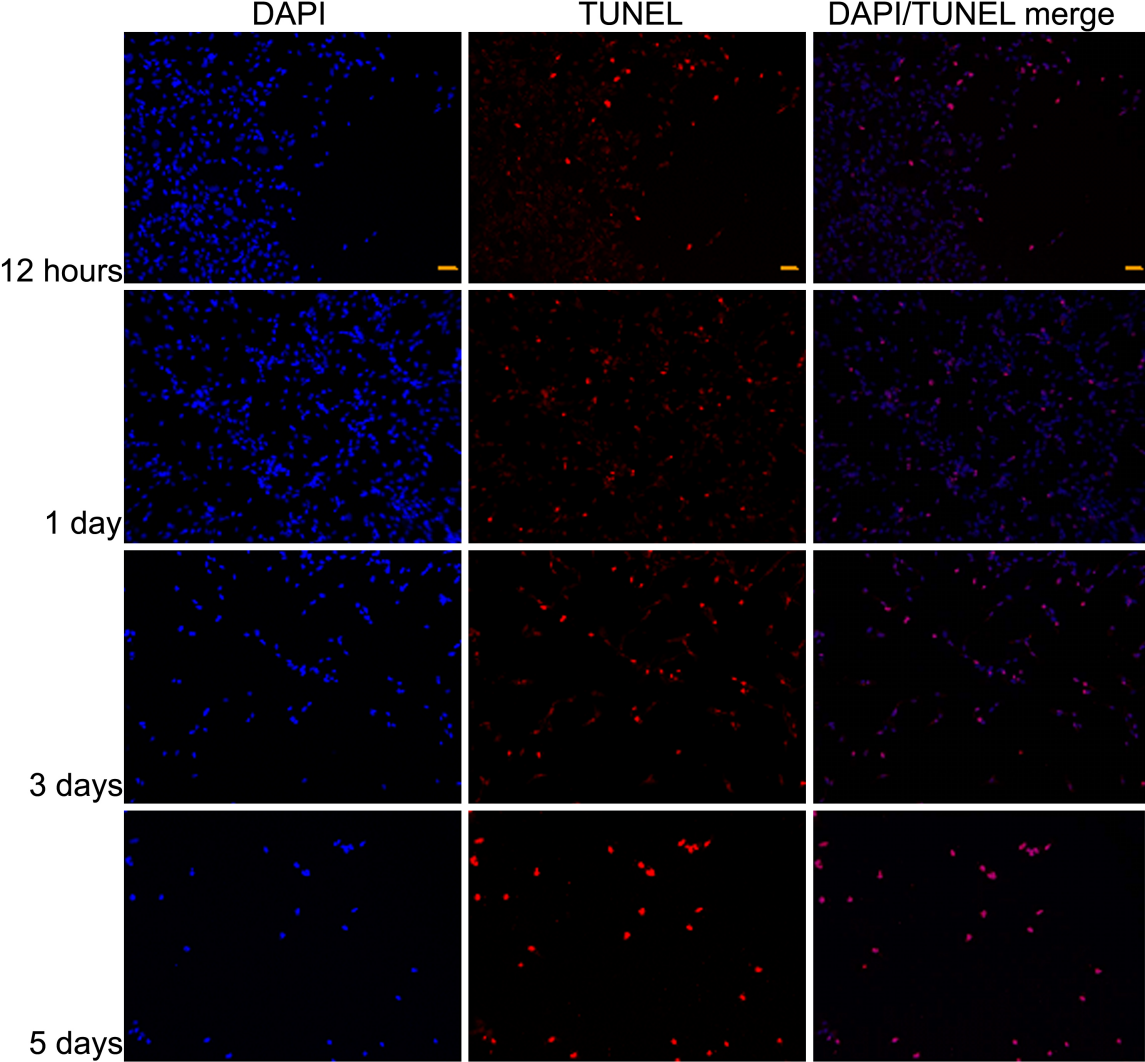
Apoptotic trabecular meshwork cells after 1 mg/ml triamcinolone acetonide treatment. Apoptotic nuclei of TM cells were detected 12 h as well as 1, 3, and 5 days after 1 mg/ml TA treatment. The slide was viewed with two filters. Stain red was viewed using a fluorescein filter to visualize end-labeled cells. Stain blue was viewed with a DAPI filter to visualize the entire cell population. Scale bar: 50 μm.

**Figure 6:**
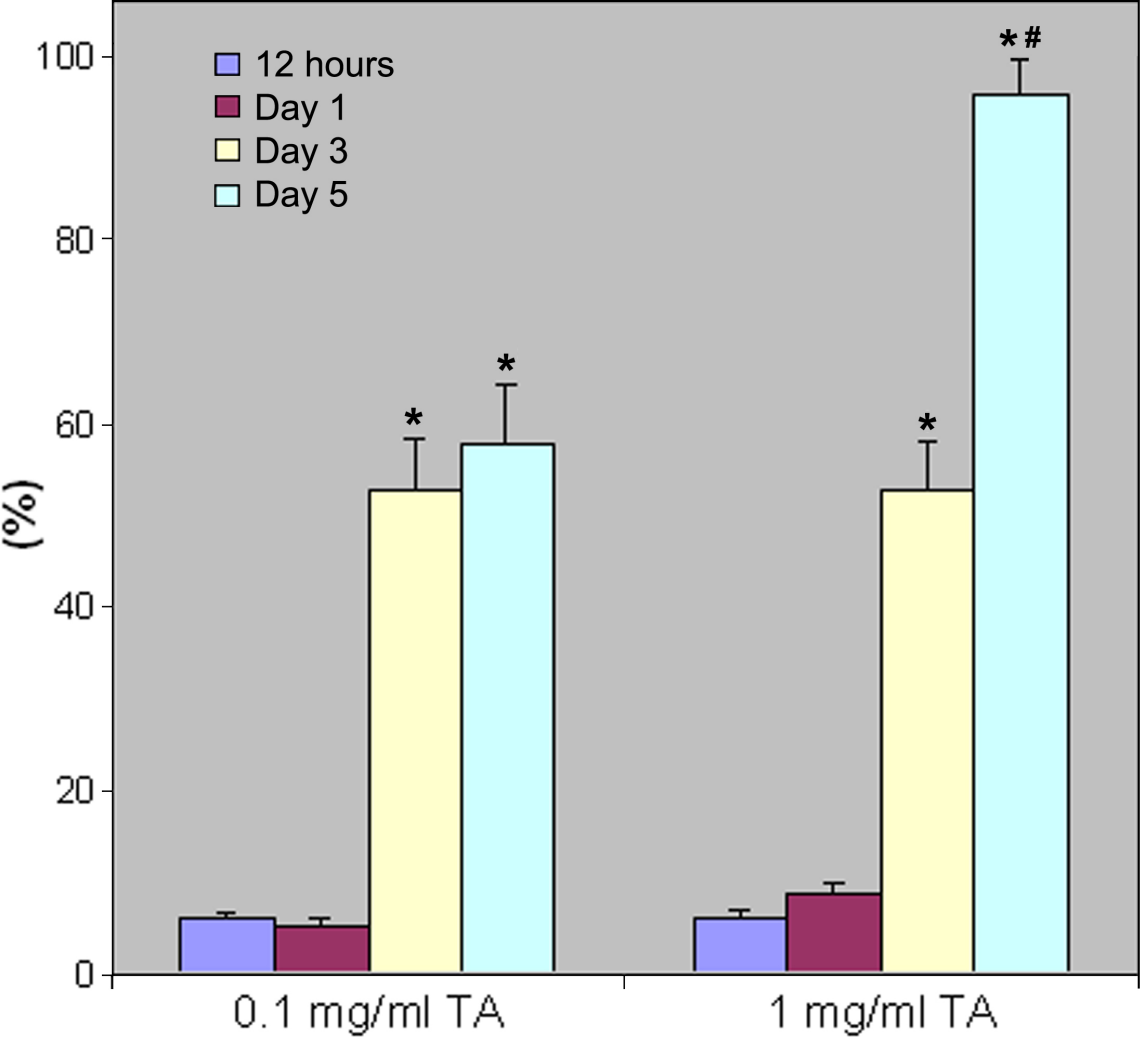
Apoptosis percentage of the trabecular meshwork cells by 0.1 mg/ml and 1 mg/ml triamcinolone acetonide treatment. The apoptosis percentage for TA, which was the apoptotic TM cell number divided by the total TM cell number, was adjusted by benzyl alcohol. The asterisk indicates that significantly increased apoptotic cells were observed with both TA treatment on days 3 and 5 compared to 12 h. The sharp (hash mark) means that when compared to 0.1 mg/ml TA treatment, the number of the apoptotic TM cells was significantly increased with 1 mg/ml TA treatment on day 5.

Apoptotic nuclei of TM cells were detected 12 h as well as one, three, and five days after treatment by both 0.1 mg/ml and 1 mg/ml TA (Figure 4,5). Significantly increased number of apoptotic cells were observed at both TA concentrations at day 3 and day 5 (Figure 6). Compared to 0.1 mg/ml TA treatment, the apoptotic TM cells were significantly increased five days after 1 mg/ml TA treatment.

## Discussion

Results of the present study showed that TM cells exposed to TA exhibit reduced cell proliferation and increased expression of genes involved in stress and apoptosis. In view of a significant difference in the number of TM cells between different concentrations of benzyl alcohol given to the culture, we eliminated the benzyl alcohol effect when we analyzed the concentration and time effects of TA treatment. Benzyl alcohol has been reported to cause a reduction in relative bodyweight, histopathologic lesion, and neurotoxicity [24]. Since benzyl alcohol is a vehicle of TA, both may cause cytotoxicity to TM cells after intraocular injection of TA. Detailed statistical analysis of the initial differential expressions of all five studied genes (Figure 3, dotted lines) revealed that significant differences persisted over several time points for all the genes (Table 1). There was also a difference in expression between 0.0025% and 0.025% benzyl alcohol. Therefore, the effects of benzyl alcohol were subtracted from the total effects of TA in benzyl alcohol (Figure 3, solid lines) which subsequently showed the sole effects of TA on the gene expressions.

It is known that in patients with glaucoma, a significant loss of TM cells occurs with increasing age. The cell depletion may lead to disintegration and loss of the trabecular beams, and the change of cell activities may adversely affect aqueous outflow, causing IOP elevation [25,26]. Whereas the changes in MTT absorbance in this study indicated the degree of necrosis, the gene expression data showed the extent of apoptosis of TM cells in the presence of TA. Apoptosis has a critical role in development, homeostasis, wound healing, and pathophysiology of diseases. It has been implicated in these processes in the retina, lens, cornea, TM, optic nerve, and the central nervous system pathways that contribute to vision [27]. Apoptosis pathways are not only stimulus-specific but also cell-type specific. Low concentrations of chemical-induced oxidative stress can elevate gene expressions of *c-jun* and *c-fos*, which protect the cells against toxic insult and enhance cell survival whereas high concentrations can lead to apoptosis with the activation of the *caspase-3* pathway [28]. Similar events might have been observed here. Significant increases in *c-fos* and *c-jun* expressions were found early in 1 mg/ml TA treatment, but the duration was longer in 0.1 mg/ml TA treatment (Figure 3A,B). Recently, *c-jun* was found upregulated in glaucoma and optic nerve transaction models [29], providing evidence of the involvement of *c-jun* NH_2_-terminal kinase as a signaling molecule and of the participation of tumor necrosis factor alpha in glaucoma.

The expression of *caspase 3* was higher in 1 mg/ml TA but only significantly different at 50 min (Figure 3D). Meanwhile, *caspase 3* was upregulated for the three types of cultured human cell lines tested for TA effects, TM, ARPE19, and SVG [18], showing a cellular sensitivity of these cultured cells on *caspase 3*. It also indicated that *caspase 3* has a major role in TA stimuli.

c-myc is a multifaceted protein that regulates the cell cycle and cell growth, enhances genomic instability, and stimulates angiogenesis, cell transformation, and apoptosis [30,31]. The main physiologic function of p53 is thought to be a cell cycle regulator at the G1 checkpoint and an inductor of apoptosis [26]. We found both c-myc and p53 upregulated by TA. However, compared to c-myc, the change in gene expression of p53 across the time points was milder and not significant until 50 min after TA treatment (Figure 3C,E). These results indicate that the TA effect on TM cells involves cell growth regulation and proliferation in contrast to RPE cells where TA affected no change in the expressions of *c-myc* and *p53* [18].

Different facets of apoptosis have been investigated: time progression, extrinsic versus intrinsic pathways, and compartmentalized occurrence in individual organelles such as the nucleus, cytosol, mitochondria, and membranes [32]. Various microscopic technologies, electron microscopy (EM), and fluorescence microscopy that involve advanced staining techniques reveal cell death and DNA fragmentations [32]. The TUNEL assay is especially useful for in situ testing for apoptosis although it is not specific for apoptosis and shows DNA cleavage from any form of cell death and even necrotic cells [33]. In our study, we confirmed the apoptosis of TM cells caused by TA treatments by the TUNEL assay. Further study can be comprehensively performed through assaying for antibodies against a target of executioner caspase and specific mediators of apoptosis.

In summary, this study is the first report on TA effects on human TM cells. Our data suggested that the use of TA may disrupt the TM function because of TA cytotoxicity on the TM cells and the elevation of stress genes, *c-fos* and *c-jun*, and of apoptosis genes, *caspase 3* and *c-myc*. This may lead to impaired aqueous outflow through the TM and subsequently, an elevation in IOP.
